# Iodinated Contrast Media Dose Protocols for Computed Tomography Investigations of the Abdomen: A Systematic Review and Meta-Analysis

**DOI:** 10.3390/jimaging12070301

**Published:** 2026-07-06

**Authors:** Evans Ohemeng, Andrew Donkor, Ijeoma Chinedum Anyitey-Kokor, Obed Kojo Otoo, Theophilus N. Akudjedu, William Kwadwo Antwi, Yaw Amo Wiafe

**Affiliations:** 1 Department of Medical Imaging, Faculty of Allied Health Sciences, Kwame Nkrumah University of Science and Technology, PMB UPO, Kumasi, Ghana; 2 IMPACCT (Improving Palliative, Aged and Chronic Care through Clinical Research and Translation), Faculty of Health, University of Technology Sydney, City Campus, 15 Broadway, Ultimo, Sydney, NSW 2007, Australia; 3 Komfo Anokye Teaching Hospital, P.O. Box 1934, Kumasi, Ghana; 4 Institute of Medical Imaging & Visualisation, Department of Medical Science & Public Health, Faculty of Health & Social Sciences, Bournemouth University, 12 St Pauls Ln, Bournemouth BH8 8GP, UK; 5 Department of Radiography, School of Biomedical and Allied Sciences, P.O. Box LG 143 Korle-Bu, Accra, Ghana

**Keywords:** abdominal imaging, iodinated contrast media, dosing protocols, organ enhancement, sustainability, potential material savings

## Abstract

While several dosing protocols for iodinated contrast media (ICM) exist, a consensus strategy for optimising the physical imaging signal in abdominal CT is lacking. This systematic review and meta-analysis evaluated the performance of individualised dosing protocols, specifically focusing on technical signal optimisation, clinical safety, potential material savings, and environmental sustainability. Electronic databases (Cochrane, Embase, Medline) were searched up to January 2026. Systematic synthesis of 23 studies (11,680 participants) compared protocols based on lean body weight (LBW), total body weight (TBW), fixed volume (FV) and software-assisted dosing. Meta-analyses assessed volume optimisation and hepatic enhancement, with evidence certainty evaluated via the GRADE framework. TBW-based dosing significantly reduced contrast volume by −8.74 mL compared to FV protocols (*p* = 0.02), while the −4.04 mL reduction in LBW versus TBW groups represented a non-significant trend (*p* = 0.11); however, a sensitivity analysis revealed a significant effect (−5.41 mL, 95% [CI: −10.43, −0.39]; *p* = 0.03). Pooled hepatic enhancement showed no statistically significant differences for LBW vs. TBW (−1.36 HU, *p* = 0.43) or FV vs. TBW (−2.74 HU, *p* = 0.13). Individualised ICM dosing, particularly LBW-based, may potentially offer a foundational strategy for greener and material savings in clinical radiology by minimising population-level iodine load. Despite modest individual volume reductions, these protocols may potentially facilitate standardised imaging enhancement, though higher-quality randomised trials are required to confirm safety and economic benefits.

## 1. Introduction

Multi-detector computed tomography (MDCT) plays a critical role in detecting several diseases, including abdominal pathologies [1]. The advantages of MDCT technology include thinner slice thickness, high temporal and spatial resolutions, fast acquisition times, lower radiation doses, high diagnostic reliability, and efficient and flexible use of contrast medium (CM) [1,2,3]. The administration of an iodine-based CM increases the visibility of vascular structures and organs during abdominal CT procedures [4]. However, the use of ICM may be associated with reactions such as nausea, vomiting, headache, itchiness, skin rashes, and fever [5].

Optimal contrast enhancement for abdominal CT has been the subject of various studies [6,7]. The factors that affect ICM enhancement in abdominal CT imaging can be grouped into technical and patient-related factors [1]. Identified technical factors include CM concentration, injection rate, duration, volume, and the use of a saline flush [7,8,9]. The most important patient-related factor is body weight, which is inversely related to the magnitude of diagnostic image enhancement [8,10,11]. Other patient-related factors include cardiac output, renal function, location of cannula, venous access, gender, and age [7,8].

Several dosing protocols for ICM have been developed in the last three decades to obtain contrast-enhanced diagnostic information for CT patients. These dosing protocols for ICM relate to fixed volume (FV), total body weight (TBW), lean body weight (LBW), body surface area (BSA), and body mass index (BMI). However, there is no consensus on one strategy. Thus, a standard protocol of optimal performance irrespective of settings is lacking. Numerous reports have been published supporting the benefits of different dosing protocols for ICM in abdominal CT [12,13]. A recent systematic review provided evidence that LBW protocols reduce contrast volume; however, that study was limited by a small cohort and did not address potential economic or safety implications. The present study fills these gaps by synthesising data from 23 studies and evaluating the technical, clinical, cost, and safety implications of dosing protocols [14]. The present review is unique as it synthesises the most recent evidence available up to 2026, encompassing a larger cohort that allows for a more definitive meta-analysis of dosing volumes and hepatic enhancement.

Furthermore, it is the first to integrate the environmental sustainability agenda into ICM optimisation, framing individualised dosing as a strategy for ‘greener’ clinical radiology. Thus, an updated systematic review and meta-analysis are warranted to provide comprehensive evidence on the technical, clinical, cost and safety implications of different ICM dosing protocols in abdominal CT. This systematic review and meta-analysis will have important clinical implications for the optimisation of ICM administration in abdominal CT imaging. By synthesising the available evidence on different dosing strategies, the review will provide a clearer understanding of how contrast protocols influence organ enhancement, image quality and diagnostic performance. This review specifically addresses the optimisation of contrast administration protocols as a foundational step in abdominal imaging. While we evaluate the resulting image quality (e.g., HU and SNR), the review does not encompass post-acquisition methods such as image reconstruction, segmentation, or automated interpretation, which remain independent areas of investigation. The findings are expected to inform more standardised yet individualised dosing practices that improve the consistency of abdominal organ enhancement and lesion detectability.

## 2. Materials and Methods

This meta-analysis was conducted and reported in accordance with the Preferred Reporting Items for Systematic Reviews and Meta-Analyses (PRISMA) statement [15]. The PRISMA checklist is included in the supplementary materials (Supplementary Table S6).

### 2.1. Data Source and Search Strategy

A comprehensive search was conducted for relevant studies in the following electronic databases: Cochrane Library (Supplementary Table S2), Embase and Medline. An additional search was performed manually using Google Scholar for relevant publications related to the subject matter. To ensure that no relevant research evidence was left out, the reference lists of the relevant literature were also scrutinised for other published works on the subject.

Databases were searched between 28 October and 2 November 2025. To ensure a comprehensive and up-to-date analysis, databases were updated as recently as 15 January 2026, capturing the most contemporary evidence in the field. The search strategy included terms relating to the following concepts: CT scan, abdomen, iodinated contrast media, radio-opaque media, contrast agent and contrast dosing protocol. Medical subject headings, keywords, and free text terms were combined using the Boolean operators “AND” or “OR.” The initial search strategy was developed in the Cochrane Library and adapted for other databases.

### 2.2. Inclusion and Exclusion Criteria

Primary studies published from 2000 onwards, of any design, that reported on dosing protocols for ICM in abdominal CT scans and were published in peer-reviewed journals in English were considered. Editorials, opinion pieces, comments, letters and reviews were excluded. The review question and inclusion criteria were structured using the PICO framework [16].

### 2.3. Study Selection

**Figure 1:**
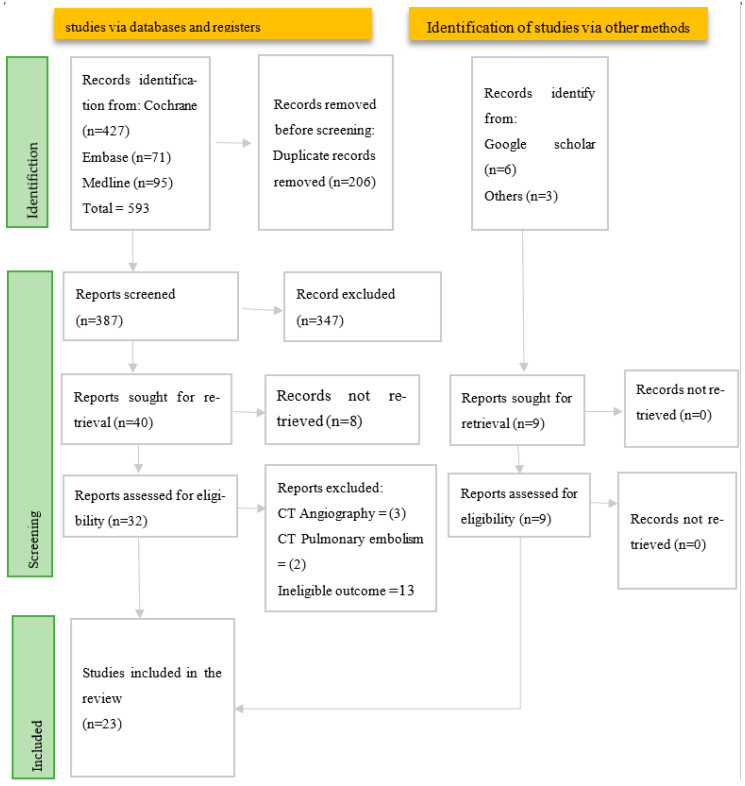
PRISMA flow diagram.

All identified citations were exported to the EndNote version X9 reference manager for duplicate removal and storage following the database search. Two authors (EO and AD) independently screened the titles and abstracts of the articles based on the eligibility criteria. Disagreements were resolved through discussion. Reasons for the inclusion or removal of articles are summarised in the PRISMA flow diagram (see Figure 1).

### 2.4. Data Extraction

A data extraction form was developed, and three authors (EO, AD, and YAW) performed full-text data extraction. The data extracted included the authors’ names, publication year, country, study aims and design, mean age, participants, gender, equipment type, mean volume of contrast administered, mean hepatic enhancement, and enhancement variability.

### 2.5. Study Quality Assessment

Two authors (EO and IA-K) independently assessed the quality of the included studies, followed by discussion among the wider research team. Randomised controlled trials (RCTs) were assessed using the Critical Appraisal Skills Programme (CASP) RCT checklist. Cohort studies were assessed using the CASP cohort checklist. Cross-sectional studies were also assessed with the CASP cross-sectional studies checklist.

### 2.6. Meta-Analysis and Certainty of Evidence

Random-effects meta-analyses were used to analyze the collected data with 95% confidence intervals (CIs). Overall group means and standard deviations were recalculated using weighted averages of subgroup means and pooled standard deviations for studies that reported outcomes separately for subgroups. Similarly, contrast volume was calculated from the extracted data on iodine dose and iodine concentration based on the relationship: volume (mL) = total iodine load (mg I)/iodine concentration (mg I/mL). For studies that presented outcome data separately for subgroups, the overall means and standard deviations were reconstructed using subgroup sample sizes, means and standard deviations. The Cochrane Handbook recommendations were followed to estimate the sample size-weighted means and pooled standard deviations.

Heterogeneity among studies was estimated using the *I*^2^ index, with values classified as no heterogeneity (0%), low heterogeneity (25% to 50%), moderate heterogeneity (51% to 75%) and high heterogeneity (greater than or equal to 75%) [17,18]. Leave-one-out sensitivity analysis was performed to examine whether single studies had a disproportionately excessive influence. Forest plots were generated, and probability values below 0.05 were considered statistically significant. Data were analysed using Review Manager 5.4. The Grading of Recommendations, Assessment, Development and Evaluation (GRADE) framework was employed to ascertain the certainty of the evidence obtained [19].

## 3. Results

### 3.1. Study Selection

The databases’ search together yielded 593 articles, of which 206 duplicate articles were removed. The 387 remaining articles were screened by their titles and abstracts, which resulted in the removal of 347 articles using the exclusion criteria. Out of the remaining 40 articles, the full texts of eight articles were unavailable. The full text of the remaining 32 articles was assessed for eligibility, of which 14 articles met the inclusion criteria. Nine additional articles were identified through hand searches; hence, 23 articles reporting on various dosing protocols for ICM in abdominal CT were included in this review (see Figure 1).

### 3.2. Characteristics of the Included Studies

**Figure 2:**
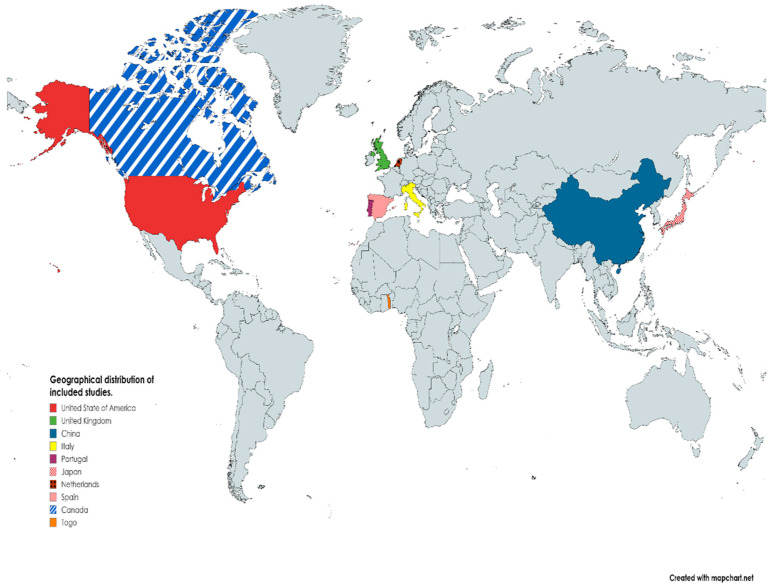
Geographic distribution of the included studies.

The baseline characteristics and geographical distribution of the included studies are shown in Table 1 and Figure 2, respectively. Detailed technical data for each study, including equipment parameters and specific contrast volumes, are provided in Supplementary Table S3. There were eleven (RCTs) [20,21,22,23,24,25,26,27,28,29,30], nine cohort studies [31,32,33,34,35,36,37,38,39] and three cross-sectional studies [40,41,42]. The total sample size for all included studies was 11,680. Participants across the included studies represented a broad clinical spectrum, including oncological patients [26,27,31,40], venous portal phase CT [41], hepatic dynamic CT [22,25,39], uniphasic CT [36], abdominal CT [20,23,24,28,33,34,35,37,38,42], and suspected abdominal disease [21,29,30,32]. The studies were conducted in Japan (*n* = 5) [20,21,22,29,39], Italy (*n* = 4) [26,27,30,40], the United Kingdom (*n* = 2) [35,38], Netherlands (*n* = 2) [31,33], China (*n* = 2) [25,32], USA (*n* = 3) [28,34,37], Canada (*n* = 2) [24,36], Portugal (*n* = 1) [41], Spain (*n* = 1) [23] and Togo (*n* = 1) [42]. Enhancement was also measured in Hounsfield Units (HU).

### 3.3. CT Scanning Procedures and Image Quality Analysis

The CT scanners used were Siemens (*n* = 7) [24,26,33,36,38,40,41], GE multi-detector CT (*n* = 10) [20,21,22,27,28,29,30,32,34,37], Philips (*n* = 2) [23,35], and Toshiba Aquilon (*n* = 1) [25]. Two studies used CT scanners from different manufacturers [31,39]. One study used three CT scanners from different centres [42]. The exposure factors used were similar in all the studies. Other parameters, such as pitch and gantry rotation time, were also similar for all studies. One study reported that the CT image quality assessment of the mean CT value in Hounsfield units (HU) of the liver was performed by a radiologist with 13 years of post-training experience [21]. All other studies used an experienced radiologist to assess CT image quality.

### 3.4. Total Body Weight-Based vs. Fixed-Volume Protocols

**Figure 3:**
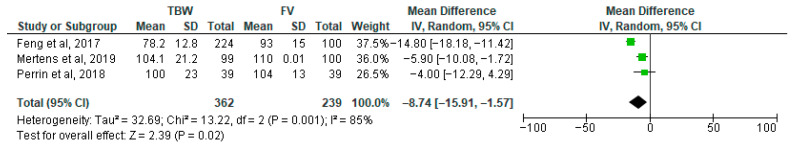
Forest plot of the volume of ICM administered using the TBW-based and FV protocol [25,33,35].

Volume of ICM administered: Three studies (2 cohort and 1 RCT) investigated the volume of ICM administered to 601 participants requiring CT scans using the TBW-based and FV protocols [25,33,35]. Figure 3 shows the mean difference in the pooled volume of ICM administered between the TBW-based and FV protocols using the random effects model. The mean difference was −8.74 mL (95% [CI: −15.91, −1.57]; *p* = 0.02). This indicates that a significantly lower volume of ICM was administered to the TBW-based protocol group than to the FV protocol group. However, a significantly high between-study heterogeneity was observed (*I*^2^ = 85%, *p* = 0.001).

**Figure 4:**
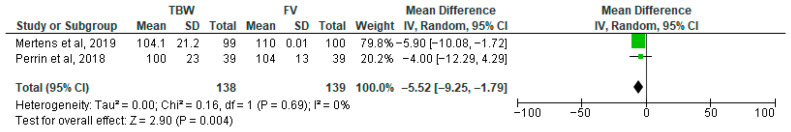
Forest plot of the volume of ICM administered using the TBW-based and FV protocols after sensitivity analysis [33,35].

Leave–one–out sensitivity analysis: When the one RCT [25] was removed through leave-one-out sensitivity analysis, the mean difference in the pooled volume of ICM administered was still significantly lower in the TBW-based protocol compared to the FV-based protocol group (−5.52 mL (95% [CI: −9.25, −1.79]; *p* = 0.004) (see Figure 4). No significant between-study heterogeneity was observed (*I*^2^ = 0%, *p* = 0.69).

**Figure 5:**
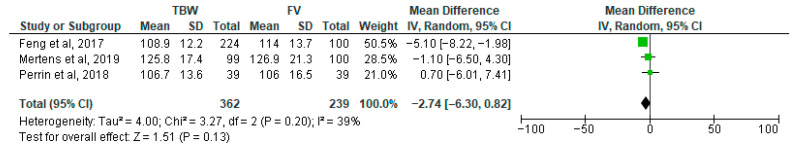
Forest plot showing the degree of hepatic enhancement using the TBW-based and FV protocols [25,33,35].

**Degree of hepatic enhancement:** Three studies (two cohorts and one RCT) investigated the degree of hepatic enhancement for 601 participants requiring CT scans using the TBW-based and FV protocols [25,33,35]. The pooled hepatic enhancement mean difference between the TBW-based and FV protocol groups was −2.74 HU (95% CI: [−6.3, 0.82]) (see Figure 5). No significant difference was observed between the two protocols (*p* = 0.13). Insignificantly low between-study heterogeneity was observed (*I*^2^ = 39%, *p* = 0.20).

**Figure 6:**
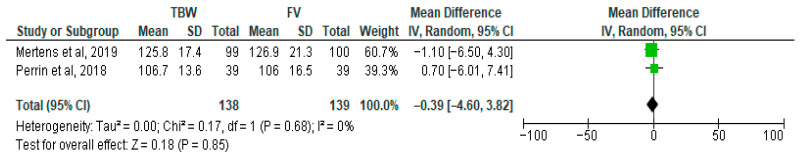
Forest plot showing the degree of hepatic enhancement using the TBW-based and FV protocols after sensitivity analysis [33,35].

Leave-one-out sensitivity analysis: When the RCT [25] was removed through leave-one-out sensitivity analysis, the mean difference in the pooled hepatic enhancement was still insignificant between the TBW and FV-based protocol (−0.39 HU (95% [CI: −4.60, 3.82]; *p* = 0.85) (see Figure 6). No significant between-study heterogeneity was observed (*I*^2^ = 0%, *p* = 0.68).

### 3.5. Total Body Weight-Based vs. Semi-Fixed Protocols

Volume of ICM administered: It was not possible to conduct a meta-analysis for the one study that compared the contrast volume to 160 participants using TBW-based and semi-fixed protocols [31]. The study identified a lower volume of ICM in the TBW-based group compared to the semi-fixed protocol group (TBW = 134 mL and SF = 140.3 mL) (*p* = 0.074).

Degree of hepatic enhancement: No meta-analysis was performed because only one study investigated the mean liver enhancement for the TBW-based and semi-fixed protocols [31]. The study identified a significant difference in mean liver enhancement between the two groups (TBW = 46.7 HU, SF = 54.2 HU; *p* < 0.001).

### 3.6. Lean Body Weight-Based vs. Total Body Weight-Based Protocols

**Figure 7:**
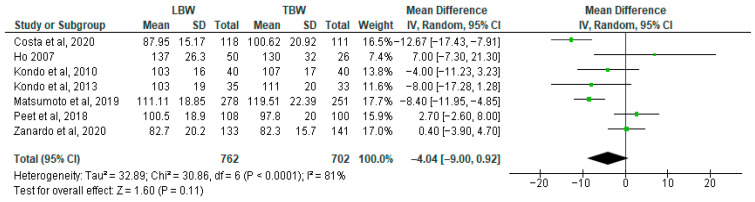
Forest plot of the volume of ICM administered using LBW-based and TBW-based protocols [21,22,24,26,28,29,36].

Volume of ICM administered: Seven studies (six RCTs and one cohort) investigated the administered volume of ICM to 1464 participants requiring CT scans using LBW-based and TBW-based protocols [21,22,24,26,28,29,36]. Figure 7 shows the mean difference in the pooled volume of ICM administered using the random effects model, which was −4.04 mL (95% [CI: −9.00, 0.92]; *p* = 0.11). This indicates an insignificantly lower administered volume of ICM to the LBW-based protocol group than the TBW-based protocol group. The between-study heterogeneity was high (*I*^2^ = 81%, *p* < 0.0001).

**Figure 8:**
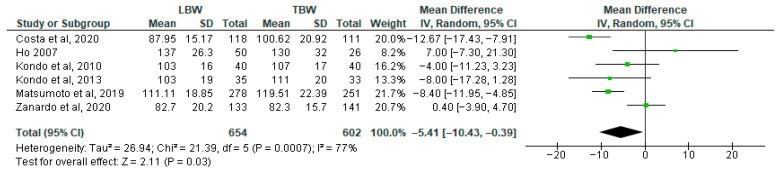
Forest plot showing the volume of ICM administered using LBW-based and TBW-based protocols after sensitivity analysis [21,22,24,26,28,29].

Leave-one-out sensitivity analysis: When the one cohort study was removed through leave-one-out sensitivity analysis [36], the mean difference in the pooled volume of ICM administered was significantly lower in the LBW-based protocol group compared to the TBW-based protocol group (−5.41 mL, 95% [CI: −10.43, −0.39]; *p* = 0.03). However, significant heterogeneity was observed (*I*^2^ = 77%, *p* < 0.0007) (see Figure 8).

Gender differences in the administered volume of ICM: One study compared the volume of ICM between females and males among the lean LBW-based and the TBW-based protocols [24]. The study reported that males received a higher volume compared to females in both protocol groups (female: LBW = 77.5 ± 11 vs. TBW = 93.7 ± 20; male: LBW = 98.4 ± 11 vs. TBW = 106.5 ± 20).

**Figure 9:**
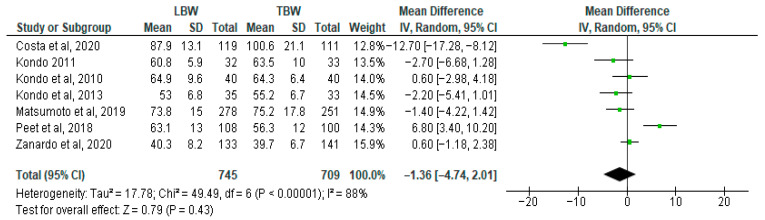
Forest plot showing the degree of hepatic enhancement using the LBW-based and TBW-based protocols [20,21,22,24,26,29,36].

Degree of hepatic enhancement: Seven studies (six RCTs and one cohort) investigated the degree of hepatic enhancement among 1454 participants requiring CT using LBW- and TBW-based protocols [20,21,22,24,26,29,36]. The pooled hepatic enhancement mean difference between the LBW- and TBW-based protocol groups was −1.36 HU (95% [CI: −4.74, 2.01]) (see Figure 9). No significant difference was observed between the two protocol groups (*p* = 0.43). High between-study heterogeneity was observed (*I*^2^ = 88%, *p* < 0.00001).

**Figure 10:**
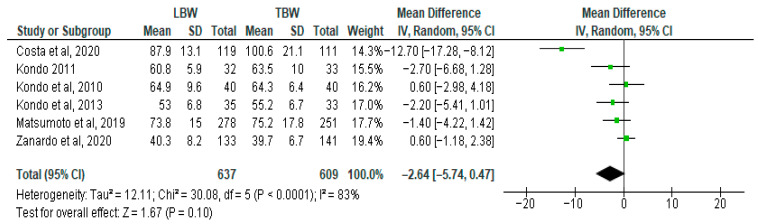
Forest plot showing the degree of hepatic enhancement using LBW-based and TBW-based protocols after sensitivity analysis [20,21,22,24,26,29].

Leave-one-out sensitivity analysis: When the single cohort study was removed through leave-one-out sensitivity analysis, the mean difference in the pooled hepatic enhancement remained insignificant between the LBW- and TBW-based protocol groups (−2.64 HU, 95% [CI: −5.74, 0.47]; *p* = 0.10). A significantly high heterogeneity was observed (*I*^2^ = 83%, *p* < 0.0001) (see Figure 10).

Gender differences in the degree of hepatic enhancement: One study compared the degree of hepatic enhancement between females and males among the LBW-based and TBW-based protocols [24]. The study reported no significant difference in the degree of hepatic enhancement between genders (female: LBW = 49.4 ± 14 vs. TBW = 54.6 ± 11; male: LBW = 51.5 ± 10 vs. TBW = 54.8 ± 11).

### 3.7. Lean Body Weight-Based vs. Fixed-Volume Protocols

**Figure 11:**
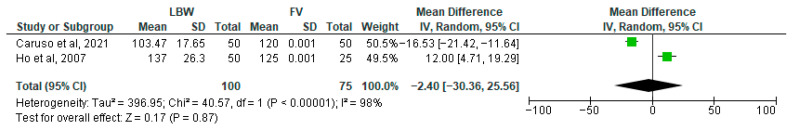
Forest plot of the administered volume of ICM using LBW-based and FV protocols [27,28].

Administered volume of ICM: Two studies investigated the administered volume of ICM to 175 patients requiring CT scans using LBW and FV protocols [27,28]. The pooled mean difference between the LBW and the FV protocol groups was −2.40 mL (95% [CI: −30.36, 25.56] *p* = 0.87) (Figure 11). This indicates no significant difference between the two protocols. The between-study heterogeneity was high (*I*^2^ = 98%, *p* < 0.00001).

Degree of hepatic enhancement: One RCT compared the mean hepatic enhancement between the LBW and FV protocols [27]. A meta-analysis could not be performed.

### 3.8. Liver Signal-to-Noise Ratio and Contrast-to-Noise Ratio

Arterial phase: It was not possible to conduct a meta-analysis because only one study reported data on liver signal-to-noise ratio and contrast-to-noise ratio in the arterial phase between the LBW-based and FV protocols [27]. There was no significant difference between the two protocols in terms of their mean signal-to-noise ratio and contrast-to-noise ratio, respectively (0.33 HU [95% CI: −0.36, 1.02]; *p* = 0.35) (−0.27 HU [95% CI: −0.78, 0.24]; *p* = 0.3). The corresponding values, however, favoured the LBW protocol in terms of contrast-to-noise ratio in the arterial phase. In contrast, the FV protocol was favoured for signal-to-noise ratio in the arterial phase.

Portal venous phase: It was challenging to perform a meta-analysis on the two studies that reported data on liver signal-to-noise ratio and contrast-to-noise ratio in the portal venous phase because they used different study designs [27,33]. One study identified an insignificant mean difference between the LBW and FV protocols in terms of contrast-to-noise ratio (0.11 HU, 95% CI: −0.54 to 0.76; *p* = 0.74) [27]. For signal-to-noise ratio, one study revealed an insignificant mean difference between the LBW-based protocol group and the FV protocol group (−0.30 HU 95% [CI: −1.12, 0.52]; *p* = 0.47) [33].

Arterial phase kidney: One study reported data on the arterial phase kidney signal-to-noise ratio and contrast-to-noise ratio between the LBW and the FV protocols [27]. The study reported a significant difference between the two protocols in terms of their contrast-to-noise ratio (−1.70 HU, 95% CI: −3.20 to −0.20; *p* = 0.03) and signal-to-noise ratio (−0.81 HU, 95% CI: −3.70 to −0.30; *p* = 0.016), favouring the LBW group [27].

### 3.9. Lean Body Weight-Based vs. Blood Volume

Administered volume of ICM: One study reported data on the volume of ICM administered among the LBW and BV groups [20]. The study found no significant difference in ICM volume between the two protocols (−0.56 mL, 95% CI: −1.5 to −0.55; *p* = 0.37).

Degree of hepatic enhancement: One study reported data on the degree of hepatic enhancement between the LBW and BV groups. The study showed no significant difference in hepatic enhancement (−0.670 HU, 95% CI: −1.12 to −0.58; *p* = 0.47) [20].

### 3.10. Lean Body Weight-Based vs. Body Surface Area

Administered volume of ICM: One study provided data on the volume of ICM between the LBW and BSA groups [21]. The study showed no significant difference between the LBW and BSA groups (−0.670 mL, 95% CI: −1.0 to −0.59; *p* = 0.47) [20].

Degree of hepatic enhancement: One study reported the degree of hepatic enhancement between the LBW and BSA groups [21]. The study revealed a stable correlation between the LBW and BSA groups (r = 0.17, *p* = 0.34).

### 3.11. Dosing Protocol Based on Multiple LBW-Based Injection Protocols

Volume of ICM administered: One study compared the ICM volume to 145 participants based on different LBW groups, namely: group A, 700 mg iodine (I)/kg of LBW; group B, 650 mgI/kg of LBW; and group C, 600 mgI/kg of LBW [30]. The study reported that patients in group A received a higher volume of ICM compared to groups B and C, respectively (see Table 2).

Degree of hepatic enhancement: The study reported that patients in group A had a greater hepatic enhancement than those in groups B and C. However, the values were not significant: −0.30 HU, 95% CI [−1.12, 0.53]; *p* = 0.46 [30].

### 3.12. Other Dosing Protocols

#### 3.12.1. Weight-Based Contrast Injector Software vs. FV Protocols

Volume of ICM administered: One study compared the ICM volume to 134 participants using weight-based contrast injector software and FV protocols; thus, a meta-analysis was not performed [34]. The study reported an insignificant mean difference in ICM volume between the weight-based contrast injector software and FV protocol groups (3.2 mL [95% CI: −1.75, 7.0]; *p* = 0.10).

Degree of hepatic enhancement: The mean hepatic enhancement was −5 HU lower in the weight-based injector software group as compared to the FV group (95% [CI: −1.75, 7.0]; *p* = 0.001).

#### 3.12.2. Dosing Protocol Based on BMI

Volume of ICM administered: One study compared the ICM volume to 126 participants based on BMI classification, namely: group 1 = BMI ≤ 24 kg/m^2^ vs. group 2 = BMI of 24.1–28 kg/m^2^ vs. group 3 = BMI ≥ 28 kg/m^2^ [32]. The study reported a mean difference in ICM volume of 40.3 mL (95% [CI: −44.4, −36.3]; *p* < 0.00001) between group 1 and group 2, as well as 76.3 mL (95% [CI: −80.46 −72.2]; *p* < 0.00001) between group 1 and group 3.

#### 3.12.3. Dosing Protocol Based on kV, BMI, Injection Rate, and Injection Duration

Volume of ICM administered: One study compared the volume of ICM administered to 218 patients based on kV ranges, BMI ranges, injection rate and duration. The mean difference in CM volume between underweight and obese patients was 52.3 mL (95% CI: 50.22, 54.45; *p* < 0.00001). The study revealed a strong negative linear correlation between contrast volume and weight [42]. The study also revealed that as kV increases, liver contrast enhancement also increases. Increasing the injection rate and duration also increased liver contrast enhancement.

Degree of hepatic enhancement: The study indicated that the mean hepatic enhancement among underweight and obese patients was 78.88 ± 17.42 HU [42].

### 3.13. Potential Financial and Material Savings

Three studies reported data on the financial burden of the use of ICM for CT scanning [23,37,40]. However, a meta-analysis could not be performed because of poor data quality. One such study reported that the use of an LBW-based protocol instead of a TBW-based protocol could impact the overall volume of ICM to the population, allowing a more tailored approach to ICM administration and a possible reduction in the total amount of ICM administered to the population [40]. The accompanying cost reduction, reliant on variations in administered volume, was another modest advantage resulting from the LBW-based dosing approach [40]. It was also reported that high cost and material savings can be modestly realised at abdominopelvic CT by adopting a weight-based dosing strategy as well as lowering CM volumes compared to a FV protocol [37]. A final study revealed that when ICM was tailored to patient weight, there was a reduction in contrast material dose, saving 0.96 gI, equivalent to about €1.34, compared to a FV protocol [23]. However, potential population-level benefits or hypothesis-generating implications would be more consistent with the evidence.

### 3.14. Potential Safety Issues

Despite the importance of ICM in CT practice for tissue differentiation, the development of side effects such as allergic-like reactions, cardiac arrhythmias, and pulmonary oedema remains possible [43]. Two studies reported having used lower doses of ICM in the LBW-based group [22,24]. Theoretically, this reduction in volume may represent an indirect advantage by lowering individualised iodine exposure; however, a reduction in clinically adverse events remains unconfirmed [40].

### 3.15. Data Quality Assessment

The methodological quality assessment of eligible studies according to the Critical Appraisal Skills Programme (CASP) checklist is shown in Supplemental Table S1. All studies had clear aims and appropriate recruitment strategies. All studies indicated that they obtained ethical clearance from recognised institutions. Five of the included studies did not identify confounding factors, nor did they identify any strategies to deal with them [37,38,39,40,41]. The majority of the cohort studies did not have long follow-ups of patients [31,32,33,34,35,37,38]. All studies used appropriate recruitment techniques.

### 3.16. Certainty of Evidence Assessment Findings

Using the GRADE framework, the certainty of evidence was judged as moderate for the comparison of TBW dosing versus FV and LBW dosing versus TBW protocols for the outcome of injected ICM volume (mean difference of −8.74 mL and −4.04 mL, respectively). For most other key outcomes (including hepatic enhancement, LBW vs. TBW comparisons, image-quality metrics, clinical safety outcomes, and economic outcomes), the certainty of evidence was low due to risk of bias, imprecision, heterogeneity, and sparse reporting (see Table 3).

## 4. Discussion

This systematic review and meta-analysis examined the various ICM dosing protocols for abdominal CT imaging. It included 23 studies, with 11,680 participants requiring abdominal CT scans. The included studies were eleven RCTs, nine cohort studies, and three cross-sectional studies. The identified ICM dosing protocols included LBW-based, TBW-based, FV-based, weight-based contrast injector software, BSA-based, semi-fixed dosing, and BMI-based dosing protocols.

Current dosing recommendations are based on TBW, which is valid for normal-weight patients [40]. The meta-analysis suggests that the administered volume of ICM was significantly higher in the FV protocol group when compared with the TBW-based protocol (−8.74 mL [95% CI: −15.25, −1.57]; *p* = 0.02). The effect was still significant when sensitivity analysis was performed. However, when the TBW-based protocol was compared with the LBW-based protocol, the administered volume of ICM was higher among participants in the TBW-based protocol group (−4.04 mL (95% [CI: −9.00, 0.92]; *p* = 0.11)). While the LBW-based protocol received a lower mean volume of ICM, this difference did not reach statistical significance, suggesting a trend rather than a definitive reduction [44]. However, when a leave-one-out sensitivity test was performed to verify the robustness of the result, the pooled effect estimates for ICM administered between the two protocols was significant, indicating the meaningful impact of LBW over TBW when homogenous study designs are applied (−5.41 mL, 95% [CI: −10.43, −0.39]; *p* = 0.03). The results of the meta-analysis suggest that the LBW-based protocol could be a more tailored protocol for adjusting the volume of ICM required for abdominal CT. While a mean reduction of 1–8 mL of ICM per patient is small, its value is multifaceted. It results in HU values that do not significantly deviate from those achieved by standard protocols, despite using lower ICM volume. Economically and ecologically, these small per-patient savings may aggregate into significant population-level benefits, including potentially reduced institutional costs and a smaller environmental footprint of radiology departments [40].

Nevertheless, the LBW-based protocol is not commonly used in clinical practice because it is time-consuming and requires calculating empirical equations, including the Boer formula or the James formula [45]. Patient demographics have changed, with obese patients seen more frequently for abdominal CT scans [28]. Dosing by the TBW-based protocol depends on linear proportion principles, which could lead to overestimation of the volume of ICM in obese patients because of poorly perfused adipose tissue, and thus lead to poor distribution of ICM [46].

The hepatic contrast enhancement resulting from the distribution of ICM is related to the iodine concentration, iodine delivery rate, and amount of administered CM [47]. The results from the meta-analysis revealed no significant difference in the degree of hepatic enhancement between the TBW-based protocol group when compared with the FV protocol (−2.74 HU [(95% [CI: −6.30, 0.82]); *p* = 0.13). Similarly, when TBW-based was compared with LBW-based, there was no significant difference in the degree of hepatic enhancement (−1.36 HU (95% [CI: −4.74, 2.01], *p* = 0.43). This result from the meta-analysis can be explained by the fact that TBW dosing involves administering excessive CM. Therefore, the advantage of transitioning to an LBW protocol is not an increase in HU, but the optimisation of the contrast signal—achieving the same diagnostic threshold while minimising the patient’s iodine exposure.

This review primarily utilised hepatic enhancement as a proxy for abdominal image quality due to its prevalence in the included studies. However, hepatic enhancement is only one component of diagnostic quality. Parameters such as enhancement of the pancreas, kidneys, and vascular structures, as well as lesion-to-background contrast, were sporadically reported and often yielded non-significant results. This highlights a critical need for future imaging research to move toward a more holistic evaluation of abdominal organ enhancement and clinical diagnostic accuracy.

While the benefits of individualised dosing apply broadly to abdominal CT, the practical advantage is most pronounced in multiphasic liver imaging and for patients with high BMI. In multiphasic liver CT, consistent contrast delivery is essential for accurate arterial and portal venous phase timing. For high-BMI patients, transitioning from TBW to LBW dosing prevents the ‘over-dosing’ effect caused by poorly perfused fat, thereby optimising the signal-to-noise ratio while reducing the potential risk of nephrotoxicity.

This systematic review highlights the potential financial and material savings of weight-based dosing protocols. Studies have suggested that adopting LBW-based dosing strategies could lead to cost savings and reduced material usage compared to FV protocols. The explanation for this is that LBW-based dosing results in a modest reduction in the total volume of ICM used per patient [6]. Future research should, however, conduct a holistic cost-effectiveness assessment. In addition to potential material savings, issues relating to patient safety were well documented in some of the included studies, although a meta-analysis could not be performed. The study revealed that LBW-based dosing may offer a strategy for minimising iodine load, which is a foundational step towards reducing the risk of nephrotoxic effects [45].

As a unique perspective offered by the current study, the findings may support the broad scheme of the environmental sustainability agenda in clinical radiology practice [48]. ICM usage has been identified as a key contributor to environmental sustainability concerns, as traces have been found in freshwater systems following patient excretion. Thus, minimal ICM usage by adopting LBW-based dosing may be considered a strategy for promoting safer and greener clinical radiology practice, though further research is needed to quantify its environmental impact.

### 4.1. Strengths and Limitations

The strengths of this study include the systematic search strategies, rigorous selection criteria, and an in-depth review process. This systematic review included studies with different study designs comparing various ICM dosing protocols in abdominal CT imaging, thereby increasing the number of studies. This approach increased the total sample size, thereby ensuring generalisation of results to support clinical practice. It was possible to compare the cost implications of tailoring ICM to patients’ weight, highlighting LBW dosing as a potentially more economical strategy. However, the review is subject to some methodological limitations. First, there was considerable heterogeneity across the included studies, particularly in terms of CT scanner models, which may independently influence image quality and contrast enhancement. Secondly, variability in contrast concentration may limit the direct comparability of outcomes across studies.

Lastly, this review relies heavily on hepatic HU values as the primary measure of performance. The lack of standardised data on diagnostic accuracy and non-diagnostic examination rates across the included studies limits our ability to conclude superiority in ‘overall’ image quality.

### 4.2. Advancing Future Research and Technological Directions

There is an urgent need for standardised, high-quality randomised controlled trials that specifically target patient-important safety outcomes and objective image-quality metrics to move evidence from low to high certainty. To overcome the time-intensive nature of manual empirical calculations, future research should prioritise anthropometric dosing integrated within automated contrast injector software. At the point of care, iodine loads can be tailored to individual body composition parameters such as LBW, BSA, BMI, blood volume and fat-free mass. These technologies can help minimise clinical workload and technical errors. Addressing these technological gaps is a critical prerequisite for transitioning individualised dosing from the research level into standardised clinical practice.

Future studies should quantify the reduction in environmental traces of ICM in water systems as a direct result of dose optimisation protocols. Lastly, studies should investigate the synergy between individualized contrast dosing and advanced image reconstruction algorithms to determine whether post-processing can further reduce the required iodine load while maintaining diagnostic confidence.

### 4.3. Novelty and Clinical Contribution

The manuscript introduces a holistic evaluation framework that moves beyond traditional technical metrics such as Hounsfield Units (HU).

A unique perspective of the present study is the alignment of contrast optimisation with the environmental sustainability agenda. The review frames minimal contrast usage as a key strategy for greener clinical radiology practice to reduce chemical traces in freshwater systems.

## 5. Conclusions

The systematic review and meta-analysis highlight the importance of tailoring ICM protocols to individual patient characteristics, particularly body composition. The clinical implications of this include potential improvements in diagnostic accuracy and patient safety. By individualising ICM, more consistent and adequate abdominal organ enhancement can be achieved, thereby increasing lesion detectability and overall diagnostic confidence. Consequently, while weight-based protocols (LBW or TBW) may modestly reduce contrast volume and ensure that organ enhancement remains within a statistically similar range to traditional protocols at a population level, current evidence is insufficient to make strong recommendations about superiority with respect to image quality or patient-important safety outcomes. This review advances the field by shifting the focus from purely technical image-quality metrics to a holistic view of potential patient safety, economic burden, and ecological impact. It provides a clearer understanding of how contrast protocols can move toward individualised practice while identifying the critical technological gaps that must be addressed to make such practices viable in daily clinical settings.

This work contributes to the field of imaging methodology by defining the boundaries of individualised dosing protocols and their impact on objective image-quality metrics. It provides the necessary technical evidence to shift toward a more predictable and standardised imaging environment.

## Figures and Tables

**Table 1:** Characteristics of the included studies.

First Author/Country	Study Design/Sampling Methods	Participants	Sample Size	Dosing Protocols Used
Caruso et al. 2025/Italy [30]	Randomised controlled trial	Patients for CECT of the liver	145	Multiple LBW-based protocols
Gbande et al. 2023/Togo [42]	Cross-sectional	Cancer patients	218	BMI vs kV vs Injection rate vs Injection duration
De Jong et al. (2022)/Netherlands [31]	Cohort	Patients for multiphasic abdominal CT	TBW = 80 SF = 80	(TBW) vs SF
Jiang et al. (2021)/China [32]	Prospective cohort	Patients with liver disease	126	BMI ranges. BMI ≤ 24 (80kV, 352 mg I) BMI 24.1–28(100 kV, 440 mgI) BMI ≥ 28 (120 kV, 550 mgI)
Caruso et al. (2021)/Italy [27]	Prospective randomised multicentre study	Cancer patients undergoing multiphasic abdominal CT	50 patients each were randomly selected and were dosed according to either lean body weight or fixed volume protocol	FV vs LBW
Costa et al. (2020)/Canada [24]	Stratified randomised controlled study	Patients for abdominal CT	229 outpatients for abdominal CT were recruited.	TBW vs LBW
Zanardo et al. (2020)/Italy [26]	Single-centre, double-blind, randomised controlled trial	Patients for Multiphasic abdominal CT	274	TBW vs LBW
Matsumoto et al. (2019)/Japan [22]	Prospective randomised study	Patients for hepatic dynamic CT	529	TBW vs LBW
Mertens et al. (2019)/Netherlands [33]	Prospective cohort	Abdominal CT patients	199	Weight-based (TBW) vs FD
Jensen et al. (2019)/. USA [34]	Retrospective cohort	Oncology patients for abdominal CT	134	Weight-based dosing (injector software) vs FV
Perrin et al. (2018)/UK [35]	Cohort study	Oncology patients for abdominal CT	39	TBW vs FV
Peet et al. (2018)/Canada [36]	Prospective cohort studies	Patients for Uniphase abdominal CT	208	TBW vs LBW
Zanardo et al. (2018)/Italy [40]	Retrospective cross-sectional study	Patients for Multiphasic abdominal CT	201	TBW vs LBW
Feng et al. (2017)/China [25]	Randomised controlled trial	Liver CT	324	Weight-based dosing (TBW) vs FD
Davenport et al. (2017)/USA [37]	Retrospective cohort	Abdominopelvic CT	6737	FV vs TBW vs LBW
George et al. (2016)/UK [38]	cohort	Abdominal CT	113	FV vs WB vs WB-reduced
Awai et al. (2016)/Japan [39]	A Prospective multicentre cohort study	Patients undergoing hepatic dynamic CT	1307 patients within the ages of 20–84.	BMI vs LBW vs BV
Kondo et al. (2013)/Japan [21]	Randomised controlled study	Patients with suspected abdominal disease	103 Patients	TBW vs LBW vs BSA
Rodrigues et al. (2013)/Portugal [41]	Prospective cross-sectional study	Patients for venous/portal phase CT	76	TBW vs LBW
Kondo et al. (2011)/Japan [29]	Randomised controlled trial	Patients with suspected abdominal disease	136	LBW vs TBW
Kondo et al. (2010)/Japan [20]	Randomised controlled trial	Abdominal CT	120	TBW vs LBW vs BV
Arana et al. (2009)/Spain [23]	Randomised parallel group study.	Abdominal contrast CT	151	WBD(TBW) vs FD
Ho et al. (2007)/USA [28]	Randomised controlled trial	Abdominal contrast CT	101	LBWm LBWc vs TBW vs FV

Abbreviations: BMI (Body Mass Index), BV (Blood Volume), CT (Computed Tomography), GE (General Electric), WBD (Weight-Based Dosing), FV (Fixed volume), SF (Semi fixed), MDCT (Multi-detector computed tomography), TBW (Total body weight), LBW (Lean body weight) and kV (Kilo volt). CE (Contrast-enhanced).

**Table 2:** Multiple LBW protocols with their corresponding volumes and hepatic enhancement.

LBW Groups	Mean Dose of Contrast Used	Mean Volume of Contrast Used (mL)	Mean Hepatic Enhancement (HU)
A	700 mgI/kg	92.3 ± 16.9	69.7 ± 15.7
B	650 mgI/kg	84.3 ± 14.6	62.4 ± 12.8
C	600 mgI/kg	79.8 ± 12.7	60.5 ± 13.1

**Table 3:** Certainty of evidence assessment findings (GRADE framework).

Outcome	No. of Studies/Participants	Relative/Absolute Effect	Certainty (GRADE)	Comments
Injected contrast volume: TBW dosing vs. FV	3 studies (*n* = 601)	MD −8.74 mL (95% CI −15.91 to −1.5–7)	Moderate	TBW dosing likely reduces injected contrast volume by ~8.74 mL compared with fixed-volume protocols. The certainty is downgraded by the possibility of a small study.
Injected contrast volume: LBW vs. TBW	7 studies (*n* = 1464)	MD −4.04 mL (95% CI −9.00 to 4.7	Moderate	LBW dosing likely reduces injected contrast by ~4.04 mL compared with TBW protocols. Clinical importance per patient is small.
Hepatic enhancement (HU): LBW vs. TBW	7 studies (*n* = 1454)	MD −1.36 HU (95% CI −4.74 to 2.01)	Low	No important difference detected; the estimate is imprecise, and confidence is limited.
Potential material savings	Limited reports	Narrative (some potential savings)	Low	Suggestive of population-level savings, but evidence is sparse and of low quality. Certainty is low because we were unable to meta-analyse the results to estimate the effect size. Results, however, are not generalisable.
Clinical safety outcomes (e.g., AKI, severe reactions)	Sparse observational reporting	Mostly observational	Low	Insufficient evidence to claim that any protocol reduces clinically important adverse events. High risk of bias.
Image quality (SNR/CNR, organ-specific)	Single-study or heterogeneous reports	Mostly non-significant differences; isolated significant findings	Low	Evidence is insufficient to determine whether there are differences in objective image-quality metrics. There is a high risk of bias.

## Data Availability

No new data were created or analyzed in this study.

## Supplementary Materials

The following supporting information can be downloaded at: https://www.mdpi.com/article/10.3390/jimaging12070301/s1. Table S1: Quality assessment, Table S2: Cochrane search, Table S3: Technical data and equipment parameters, Table S4: Comparison of present study with 2024 study, Table S5: Feasibility assessment of planned subgroup analysis, Table S6: Summary of compliance with PRISMA guidelines.

## Abbreviations

CASP	Critical Appraisal Skills Programme
ICM	Iodinated Contrast Media
CT	Computed Tomography
HU	Hounsfield Units
LBW	Lean Body Weight
MDCT	Multi-Detector Computed Tomography
PRISMA	Preferred Reporting Items for Systematic Review and Meta-Analysis
kV	Kilovolt
TBW	Total Body Weight
BMI	Body Mass Index
