# Supplementary material for: Iodinated Contrast Media Dose Protocols for Computed Tomography Investigations of the Abdomen: A Systematic Review and Meta-Analysis
Source: J Imaging. 2026 Jul 6;12(7):301. doi: 10.3390/jimaging12070301 (PMC13412522; doi:10.3390/jimaging12070301)
Supplement: Supplementary file 1 [file jimaging-12-00301-s001.zip › jimaging-4326443-supplementary.pdf]

Supplementary Tables

Supplementary Table S1: Quality Assessment

Cross-sectional studies

**CASP Checklist Questions**	**Zanardo (2018)**	**Rodriguez (2013)**	**Gbande (2023)**
Were the criteria for inclusion in the sample clearly defined?	Yes	Yes	Yes
Were the study subjects and the setting described in detail?	Yes	Yes	Yes
Was the exposure measured in a valid and reliable way?	Yes	Yes	Yes
Were objective, standard criteria used for measurement of the condition?	Yes	Yes	Yes
Were confounding factors identified?	No	No	Yes
Were strategies to deal with confounding factors stated?	No	No	No
Were the outcomes measured in a valid and reliable way?	Yes	Yes	Yes
Was an appropriate statistical analysis used?	Yes	Yes	Yes
Total % of "yes" to critical appraisal questions	75.0%	75.0%	87.5%

Cohort studies

CASP Checklist Questions	Awai (2016)	Peet (2018)	Jensen (2019)	De Jong (2022)	Jiang (2021)	Martens (2019)	Davenport (2017)	George (2016)	Perrin (2018)
Did the study address a clearly focused issue?	Yes	Yes	Yes	Yes	Yes	Yes	Yes	Yes	Yes
Was the cohort recruited in an acceptable way?	Yes	Yes	Yes	Yes	Yes	Yes	Yes	Yes	Yes
Was the exposure accurately measured to minimise bias	Yes	Yes	Yes	Yes	Yes	Yes	Yes	Yes	Yes
Was the outcome accurately measured to minimise bias	Yes	Yes	Yes	Yes	Yes	Yes	Yes	Yes	Yes
Have the authors identified all important confounding factors?	No	Yes	Yes	Yes	Yes	Yes	No	No	No

Have they taken account of the confounding factors in the design and/or analysis?	No	No	Yes	Yes	Yes	Yes	Yes	No	No
Was the follow up of subjects complete enough?	Yes	Yes	Yes	No	No	Yes	Yes	Yes	Yes
Was the follow-up of subjects long enough?	Yes	Yes	No	No	No	No	No	No	No
Do you believe the results?	Yes	Yes	Yes	Yes	Yes	Yes	Yes	Yes	Yes
Can the results be applied to the local population?	Yes	No	Yes	Yes	Yes	Yes	Yes	Yes	Yes
Do the results of this study fit with other available evidence?	Yes	Yes	Yes	Yes	Yes	Yes	Yes	Yes	Yes
Total % of “yes” to critical	81.8 %	90.9 %	90.9%	81.8 %	81.8 %	90.9%	81.8%	81.8%	90.9%

appraisal questions									

Randomised Controlled Trial

CASP Checklist Questions	Caruso (2025)	Caruso (2021)	Costa (2020)	Zanardo (2020)	Matsumoto (2019)	Feng (2017)	Kondo (2013)	Kondo (2010)	Arana (2010)	Kondo (2011)	Ho (2007
Was true randomisation used for the assignment of participants to the treatment group?	Yes	Yes	Yes	Yes	Yes	Yes	Yes	Yes	Yes	Yes	Yes
Was allocation to treatment groups concealed?	Yes	Yes	Yes	Yes	Yes	Yes	Yes	Yes	Yes	Unclear	Yes
Were the treatment groups similar at the baseline?	Yes	Yes	Yes	Yes	Yes	Yes	Yes	Yes	Yes	Yes	Unclear

Were participants blind to treatment assignment?	Yes	Yes	Yes	Yes	Yes	Yes	Yes	Yes	Yes	Unclear	Yes
Were those delivering treatment blind to treatment assignment?	Yes	Yes	Yes	Unclear	Yes	Yes	Unclear	Unclear	Yes	Yes	Yes
Were outcomes assessors blind to treatment assignment?	Yes	Yes	Yes	Yes	Yes	Yes	Yes	Yes	Yes	Yes	Yes
Were the treatment groups treated identically, other than the intervention of interest?	Yes	Yes	Yes	Yes	Yes	Yes	Yes	Yes	Yes	Yes	Yes
Was the follow-up complete, and if not, were differences between	Yes	Yes	Yes	Yes	Unclear	Unclear	Unclear	Unclear	Unclear	No	No

groups in terms of their follow-up adequately described and analysed?											
Were participants analysed in the groups to which they were randomised?	Yes	Yes	Yes	Yes	Yes	Yes	Yes	Yes	Yes	Yes	Unclear
Were outcomes measured in the same way for treatment groups?	Yes	Yes	Yes	Yes	Yes	Yes	Yes	Yes	Yes	Yes	Yes
Were outcomes measured in a reliable way?	Yes	Yes	Yes	Yes	Yes	Yes	Yes	Yes	Yes	Unclear	Yes

Was an appropriate statistical analysis used?	Yes	Yes	Yes	Yes	Yes	Yes	Yes	Yes	Yes	Unclear	Yes
Was the trial design appropriate, and were any deviations from the standard RCT design (individual randomisation, parallel groups) accounted for in the conduct and analysis of the trial?	Yes	Yes	Yes	Yes	Yes	Yes	Yes	Yes	Yes	Unclear	Yes
Total % of "yes" to critical	100 %	100 %	100 %	92.3 %	92.3%	92.3 %	84.6 %	84.6 %	92.3 %	69%	76%

appraisal questions											

Supplementary Table S2: Cochrane Search

Search No.	Description	Search Term	Results
#1		Mesh descriptor:(Contrast media) explode all trees	3819
#2		(Radio?opaque medi* OR radio?contrast medi* OR Contrast materia* OR contrast agent*	13231
#3	Combine	#1 OR #2	15115
#4		(Lean body?weight)	94
#5		(Body surface? Area)	8
#6		(BSA)	2406
#7		(Fixed?dose)	4452
#8		(Semi?fixed dose	1
#9		(Total body?weight)	5641
#10		(BMI)	70826
#11		(Dose protocol*)	39873
#12		(Dosing protocol*)	4723
#13	Combine	#4 OR #5 OR #6 OR #7 OR #8 OR #9 OR #10 OR #11 OR #12	17568
#14	Combine	#3 OR #13	32502
#15		MeSH descriptor (Tomography Scanners, X-Ray Computed) explode all trees	55
#16		(Computed tomography scan* OR CT scan* OR X?ray CT* OR X?ray computed tomography scan*	21240

		OR X?ray computed scan*)	
#17	Combine	#15 OR #16	21240
#18		MeSH descriptor (Pelvis) explode all trees	1733
#19		(Pelvis OR Pelvic region)	7643
#20		(MeSH descriptor (Abdomen) explode all trees	3903
#21		(Abdomen OR Abdominal OR Abdominopelvic	63163
#22	Combine	#18 OR #19 OR #20 OR #21	70750
#23		#14 AND #17 AND #22	427

Supplementary Table S3: Technical data and Equipment parameters

**First author/Country**	**Study aim**	**Gender**	**Mean age(years)**	**Equipment type used**	**Average dose of contrast used (g I)**	**Average volume of contrast used (mL)**	**Mean hepatic enhancement (HU)**	**Enhancement variability**

Caruso et al, 2025/Italy [30]	To evaluate liver enhancement and image quality of abdominal CECT examinations acquired with multiple LBW-based contrast medium injection protocols.	Female =64 Males= 81	A=70 ± 11 B=67 ± 11 C=66 ± 12	128 GE Revolution Evo	A, 700 mg iodine(I)/kg of LBW; B, 650 mgI/kg of LBW; and C, 600 mgI/kg of LBW	A=92.3 ± 16.9 B=84.3 ± 14.6 C=79.8 ± 12.7	A=69.7 ± 15.7, B=62.4 ± 12.8, C =60.5 ± 13.1.	-
Gbande et al, 2023/Togo [42]	To evaluate iodinated contrast media use in abdominal CT in cancer assessment	Female = 124 Male =94	50.92 ±15.78	16 Row and 64 Row CT scanners	BMI=30.29 ±0.91 kV=29.59± 1.06 Injection rate=31.00± 0.45 Injection duration=31 .00±1.37	-	78.88 ± 17.42	-
De Jong et al. (2022)/Netherlands [31]	To compare the homogeneity of liver attenuation in multiphasic CT patients using the	Male =95 Female =65	63.5± 0.00	Several CT scanners	TBW= 40.2 SF= 42.1	-	TBW= 46.7 SF = 54.2	-

	TBW against the semi-fixed protocol.							
Jiang et al. (2021)/China [32]	To evaluate an individualised liver CT protocol based on body mass index.	-	59.0 ± 12.6	64-MDCT GE	BMI A=20.4 ± 2.5 BMI B= 32.5 ± 3.1 BMI C = 43.3 ± 2.7	BMI A=68.1 ± 8.5 BMI B = 108.6 ± 10.6 BMI C = 144.6 ± 9.2	-	-
Caruso et al. (2021)/Italy [27]	To compare the performance of the total body weight and lean body weight dosing protocol in terms of image quality and parenchymal enhancement	Male = 24 Female = 26	65.78± 12.56	64-row MDCT	-	FV = 120± 0.01 LBW = 103.47±17.65±	FV: 59.61 ± 15.21 LBW: 59.22 ± 11.14	FV =55.2 LBW=53.0
Costa et al. (2020)/Canada [24]	To compare the magnitude and interpatient variability when dosing contrast	TBW group Men =60 Women =51 LBW group Men=59	55.4 ± 16.00	Siemens 64 slides	-	Female TBW=93.7 ± 20 LBW=77.5±11 Male TBW=106.5 ± 20= LBW=98.4 ± 11	Female TBW=54.6 ± 11 LBW=49.4± 14 Male TBW=54.8±11 LBW=51.5±10	-

	media by total body weight and lean body weight.	Women 59						
Zanardo et al. (2020)/Italy [26]	To assess whether an iodinated contrast agent could be more appropriately dosed on a patient's lean body weight than total body weight	Males =153 Females=121	Median age TBW =70 LBW= 66	Siemens Somatom 64	-	Median volume injected TBW=82(72-93) LBW=83(69-96) Mean volume LBW=82.7±20.2 TBW=82.3±0.00	TBW=97 LBW=97	Interquartile intervals TBW (53-60HU) LBW (51-61HU)
Matsumoto et al. (2019)/Japan [22]	To compare liver and aorta enhancement with contrast material doses based on TBW or LBW	Males =279 Females = 250	Median age= 66	64 - MDCT GE	-	LBW: Low BMI: 620.7 (593.0–652.9) Normal BMI: 558.2 (455.6–640.0) High BMI: 507.0 (392.6–581.3) TBW: Low BMI: 600.0 (600.0–607.6) Normal BMI:	Low BMI LBW: 76.9 TBW: 76.8 Normal BMI LBW: 76.2 TBW: 75.4 High BMI LBW: 68.8 TBW: 74.4	-

						600.0 (587.3–609.5) High BMI: 600.0 (584.4–609.4)		
Mertens et al. (2019)/ Netherlands [33]	Evaluate the attenuation and image quality of a body weight-adapted contrast media protocol compared to a fixed injection protocol	Male = 105 Female = 94	64.3 ± 15.3	Siemens CT	-	TBW=104.1 ± 21.2 Fixed dose=110 ±0.00	Weight based (TBW) = 125.8 ± 17.4<br><br>Fixed dose = 126.9 ± 21.3	-
Jensen et al. (2019)/. USA [34]	To evaluate enhancement quality and solid organ lesion depiction using weight-based contrast dosing calculated by the injector software versus a fixed contrast dose	Males = 66 Female = 68	59.0 ± 0.00	GE Revolution CT.	WBD (injector software) = 44.60 ± 6.25 Fixed dose = 45.73 ± 4.78	-	-	-

Perrin et al. (2018)/UK [35]	Assess contrast enhancement of the aorta, liver and spleen during abdominal CT using a weight–adapted contrast media protocol compared to the fixed dose method	Females = 15 Males = 24	60.0 ± 16.0	256 Slice Philips CT	-	TBW=100±23.0 FD=104±13.0	TBW = 106.7 ± 13.6 FD = 106.0 ± 16.5	-
Peet et al. (2018)/Canada [36]	To compare the magnitude and inter-patient variability in mean hepatic enhancement when dosing contrast according to total body weight versus lean body weight in a Western	Male =91 Female =117	58.5±16.00	Siemens Definition 64	TBW= 36.2 ± 7.4 LBW =37.2 ± 7.0	TBW=97.8±20 LBW=100.5±18.9	TBW=56.3±12 LBW=63.1±13	-

	population							
Zanardo et al. (2018)/Italy [40]	To report on the experience of dosing according to lean body weight	Males= 106 Females=95	66.00± 13.00	Siemens Somatom Definition 64	TBW = 33.1± 0.88 LBW = 33.3± 0.99	TBW = 89.4 ± 2.4 LBW = 90± 2.7	-	-
Feng et al. (2017)/China [25]	To investigate the effectiveness of a new individualised contrast medium injection protocol for enhanced liver CT.	-	50.5 ± 11.85	Toshiba Aquilion 64	WBD = 23.48 ± 3.84 Fixed dose = 27.9 ± 4.5	WBD=78.3±12.8 Fixed dose= 93±15	WBD = 108.9 ± 12.2 FD = 114.0 ± 13.7	-
Davenport et al. (2017)/USA [37]	To determine the magnitude of subject-level and population-level cost savings that could be realised by moving from fixed volume	Males = 3541 Females= 3196	57.8 ± 15.4	Variety of Lightspeed64-Slice CT GE	F7D= 46.2 (370Gi) vs TBW= 40.9 vs LBW =39.9	-	-	-

	low-osmolarity iodinated contrast to an effective weight–based protocol							
George et al. (2016)/UK [38]	To compare a fixed-dose intravenous iodinated contrast medium protocol with weight-based dosing protocols for abdominal CT	Not reported	-	128-Siemens Somatom	Fixed dose = 29.4<br>Vs<br>WB = 27.12 ± 5.52<br>Vs<br>WB-reduced = 18.03 ± 5.52	Fixed dose =Ideal enhancement (39)<br>Vs<br>WBD = (58)<br>Vs<br>WBD-reduced = (7)	-	-
Awai et al. (2016)/Japan [39]	To identify the body size parameter that exhibits the best correlation with aortic and hepatic enhancement at hepatic dynamic CT	Male= 818<br>Female = 489	67.0±0.00	54 CT Scanners from different manufacturers	BMI = 15.6± 2.3<br>LBW = 31.5 ± 5.2<br>BW =40.3 ± 7.3	-	-	-

Kondo et al. (2013)/Japan [21]	To compare LBW, TBW and BSA for the adjustment of iodine dose required for contrast enhanced CT	Male = 57 Female = 46	62.9±9.8	GE MDCT 16	-	TBW= 111 ± 20 LBW = 103 ± 19 BSA = 113 ± 15	TBW=55.2±6.7 LBW=53.0±6.8 BSA= 59.1 ±9.4	-
Rodrigues et al. (2013)/Portugal [41]	To verify whether the volume of intravenous CM in abdominal CT, calculated based on lean body weight, allows good liver enhancement	Male =46 Female = 30	62.42± 15.50	Siemens Somatom Sensation 40	-	-	-	TBW=53.77 LBW=-25.50
Kondo et al, (2011)/ Japan [29]	To determine the optimal iodine dose for aortic and hepatic enhancement at MDCT by	-	-	GE Lightspeed I6	-	-	550 mgI/kg LBW:43.1 ±6.0 650 mgI/kg LBW: 55.4 ±7.6 750 mgI/kg LBW: 60.8 ±5.9 600	-

	comparing lean body weight and total body weight						mgI/kg TBW: 63.5 ±10.	
Kondo et al. (2010)/Japan [20]	To evaluate and compare TBW, LBW and estimated blood volume BV for the adjustment of iodine dose required for contrast - enhanced multidetector CT of the aorta and liver	Female = 66 Male = 54	64.1	MDCT GE	TBW =32.1 ± 5.1 Vs LBW = 30.9 ± 4.8 Vs BV = 31.5 ± 4.8	TBW = 107 ± 17 Vs LBW = 103 ± 16 BV = 105 ± 16	TBW = 64.3 ± 6.4 Vs LBW = 64.9 ± 9.6 BV = 63’3 ± 5.9	-
Arana et al. (2009)/Spain [23]	To analyse the influence of contrast dose adjusted by weight vs fixed contrast dose in the	Females = 77 Males =74	55.5 ± 16.0	Philips CT scanner	-	WBD (TBW) 100.00 Vs Fixed dose = 120	-	-

	attenuation and cost of abdominal CT							
Ho et al. (2007)/ USA [28]	To prospectively evaluate the use of lean body weight (LBW) as the main determinant of the volume and rate of contrast material administration during multi–detector row computed tomography of the liver	Females=45 Males=56	53 ± 0,00	GE Lightspeed	-	LBWm =135 ± 28 LBWc=139±25 TBW= 130±32 FV=125±0	LBWm =120 ± 11 LBWc= 121±14 TBW= 124±14 FV=116 ±15	-

Supplementary Table S4: Comparison of the present study with Gulizia et al. (2024).

Feature	Gulizia et al. (2024) [Reference 14]	Present Study
Search timeline	Included studies up to 2024.	Updated search up to January 2026
Number of included studies	8 studies	23 studies
Study designs	RCTs	Eleven RCTs, nine cohort studies, and three cross-sectional studies
Total sample size	1,522 participants	11,680 participants
Intervention vs comparator	• LBW vs TBW<br>• LBW vs FV<br>• LBW vs Blood volume<br>• LBW vs BSA	• LBW vs TBW<br>• LBW vs FV<br>• LBW vs Blood volume<br>• LBW vs BSA<br>• TBW vs FV<br>• TBW vs SF

		• Weight-based contrast injector software vs FV<br>• Other protocols (multiple LBW-based injection protocols and a protocol based on kV, BMI, injection rate and injection duration)
Primary outcomes	• Volume of iodinated contrast media administered<br>• Degree of hepatic enhancement	• Volume of iodinated contrast media administered<br>• Degree of hepatic enhancement<br>• Gender differences in administered contrast volume and hepatic enhancement
Main findings		
LBW vs TBW		
• Liver parenchyma enhancement	No significant difference; pooled mean difference = −1.50 HU (95% [CI: -3.12 – 0.12]; *p* = 0.07), I^2 = 41%	No significant difference; pooled mean difference = -1.36 HU (95% [CI: -4.71, 2.01]; *p* = 0.43), I^2 = 88%
• Contrast volume	Significant difference; pooled mean difference = -7.29ml (95 % [CI: -12.04 – -2.54]; *p* = 0.003), I^2 = 79%	No significant difference; pooled mean difference = -4.04ml (95% [CI: -9.00, 0.92]; *p* = 0.11), I^2 = 81%
	Sensitivity analysis not performed	A sensitivity test showed a significant ICM volume mean difference between the two protocols -5.41ml (95% [CI: -10.43, -0.39]; *p* = 0.03), I^2 = 77%
LBW vs FV		
• Liver parenchyma enhancement	Meta -analysis could not be performed	Meta -analysis could not be performed
• Contrast volume	No significant difference; pooled mean difference = −2.40ml (95 % CI: −30.36 – 25.56]; *p* = 0.87), I^2 = 98%	No significant difference; pooled mean difference = -2.40ml (95% [CI: -30.36, 25.56]; *p* = 0.87) I^2 = 98%
LBW vs blood volume		
• Liver parenchyma enhancement	Meta-analysis not performed; a single study	Meta-analysis not performed; a single study
• Contrast volume	Meta-analysis not performed; a single study	Meta-analysis not performed; a single study
LBW vs BSA		
• Liver parenchyma enhancement	Meta-analysis not performed; a single study	Meta-analysis not performed; a single study
• Contrast volume	Meta-analysis not performed; a single study	Meta-analysis not performed; a single study
TBW vs FV		
• Liver parenchyma enhancement	Not reported	No significant difference between the two protocols, mean difference

		= (-2.74HU (95% [CI: -6.30, 0.82]; *p* = 0.13).
• Contrast volume	Not reported	There was a significant difference between the two protocols, with a mean difference of (-8.74ml (95% [CI: -12.29, -1.57.0]; *p* = 0.02). I^2 = 85%
	Sensitivity analysis not done	A sensitivity test showed a significant ICM volume mean difference between the two protocols (-5.52ml, 95% [CI: -9.25, -1.79]; *p* = 0.004), I^2 = 0%
TBW vs SF		
• Liver parenchyma enhancement	Not reported	Significant difference between the two protocols: TBW=46.7HU, SF=54.2HU p < 0.001
• Contrast volume	Not reported	Identified a lower ICM volume between the two protocols TBW=134ml, SF=140.3ml p=0.074
Injector software vs FV protocols		
• Liver parenchyma enhancement	Not reported	Significantly lower mean hepatic enhancement in the weight-based injector software group (5HU; *p* = 0.001).
• Contrast volume	Not reported	No significant difference between the weight – based and injector software protocols; mean difference = (3.2ml (95% [CI: -1.75, 7.0]; *p* = 0.10).
Dosing protocol based on kV, BMI, injection rate and injection duration		
• Liver parenchyma enhancement	Not reported	Observed mean hepatic enhancement between underweight and obese patients was 78.88 ± 17.42 HU
• Contrast volume	Not reported	Significant difference observed between underweight and obese patients; mean difference = 52.3mI (95% [CI:50.22, 54.45]; *p* <0.00001
**Other findings**		
Arterial phase		
• Liver signal-to-noise ratio	Not reported	No significant difference observed in mean HU between the LBW and FV protocols: mean difference = (0.33 HU, 95% [CI: -0.36, 1.02]; *p* = 0.35)
• Contrast-to-noise ratio	Not reported	No significant difference observed in mean HU between the LBW and FV protocols: mean difference = -0.27 HU 95% [CI: -0.78, 0.24]; *p* = 0.3).

Portal venous phase		
• Liver signal-to-noise ratio	Not reported	No significant difference observed in mean HU between the LBW and FV protocols: mean difference = -0.30 HU 95% [CI: -1.12, 0.52]; *p* = 0.47)
• Contrast-to-noise ratio	Not reported	No significant difference observed in mean HU between the LBW and FV protocols: mean difference = 0.11 HU 95% [CI-0.54, 0.76]; *p* = 0.74)
Arterial phase kidney		
• Signal-to-noise ratio	Not reported	Significant difference observed between the LBW and FV protocols. mean difference -0.81 HU 95% [CI: -3.70, -0.30]; *p* = 0.016)
• Contrast-to-noise ratio	Not reported	Significant difference observed between the LBW and FV protocols. mean difference = -1.70 HU 95% [CI: -3.20, -0.20]; *p* = 0.03)
Potential financial and material savings	Not reported	Tailoring contrast media dose to patient weight reduced contrast media use by 0.96 gI and saved approximately €1.34 per examination compared with fixed-volume dosing
Potential safety issues	Not reported	LBW-based dosing reduced media volume and iodine exposure, but its effect on reducing adverse events remains unproven

Supplementary Table S5: Feasibility assessment of planned subgroup analysis

Protocol	Outcome	Subgroup Variable	Feasible (Yes/No)	Justification
**LBW vs TBW**				
	• Contrast enhancement<br>• Contrast volume	Study design (RCT vs cohort studies)	No	Subgroup meta-analysis requires multiple studies within each subgroup. Six studies were RCTs, and one was a cohort study. Hence, there were insufficient studies per design category, preventing reliable estimation of subgroup effects. [21,22,24,26,28,29,36]

	Gender (male vs female)	No	A single study provided sex-specific outcome data, which was inadequate to perform subgroup analysis. [24]
	Age groups	No	No included study reported age-stratified outcome data, which prevented subgroup comparisons.
	BMI (normal weight, overweight and obese)	No	No included study reported BMI-stratified outcome data, which prevented subgroup comparisons.
	CT examination type (hepatic, oncologic, routine, multiphasic, uniphasic and venous /portal phase CT)	No	There were variations in examination types, with inadequate numbers of studies within each category for subgroup comparisons.
**LBW vs FV**			
• Contrast enhancement<br>• Contrast volume	Study design (RCT vs cohort studies)	No	Only two RCTs reported data, which were inadequate for subgroup comparisons. [27,28]
	Gender (male vs female)	0	No included study reported sex-specific outcome data, which prevented subgroup comparisons.
	Age group	0	No included study reported age-stratified outcome data, which prevented subgroup comparisons.
	BMI (normal weight, overweight and obese)	0	No included study reported BMI-stratified outcome data, which prevented subgroup comparisons.
	CT examination type (hepatic, oncologic, routine, multiphasic, uniphasic and venous /portal phase CT)	0	Inadequate number of studies within each category for subgroup comparisons.
**LBW vs blood volume**			
• Contrast enhancement<br>• Contrast volume	Study design (RCT vs cohort studies)	No	A single study provided outcome data, which prevented subgroup analysis.[20]
	Gender	No	No included study reported sex-specific outcome data, which prevented subgroup comparisons.
	Age group	No	No included study reported age-stratified outcome data, which prevented subgroup comparisons.

		BMI (normal weight, overweight and obese)	No	No included study reported BMI-stratified outcome data, which prevented subgroup comparisons.
		CT examination type (hepatic, oncologic, routine, multiphasic, uniphasic and venous /portal phase CT)		A single study provided outcome data, which prevented subgroup analysis.[20]
**LBW vs BSA**				
	Contrast enhancement	Study design (RCT vs cohort studies)	No	A single study provided outcome data, which prevented subgroup analysis.{21]
		Gender	No	No included study reported sex-specific outcome data, which prevented subgroup comparisons.
		Age group	No	No included study reported age-stratified outcome data, which prevented subgroup comparisons.
		BMI (normal weight, overweight and obese)	No	No included study reported BMI-stratified outcome data, which prevented subgroup comparisons.
		CT examination type (hepatic, oncologic, routine, multiphasic, uniphasic and venous /portal phase CT)		A single study provided outcome data, which prevented subgroup analysis.[21]
**TBW vs FV**				
	Contrast enhancement	Study design (RCT vs cohort studies)	No	Four studies provide data [25,33,35,37]. There were not sufficient studies per design category, which prevented reliable estimation of subgroup effects.
		Gender	No	No included study reported sex-specific outcome data, which prevented subgroup comparisons.
		Age group	No	No included study reported age-stratified outcome data, which prevented subgroup comparisons.
		BMI		No included study reported BMI-stratified outcome data, which prevented subgroup comparisons.
		CT examination type	No	There were variations in examination types, with inadequate numbers of

				studies within each category for subgroup comparisons.
**TBW vs SF**				
	Contrast enhancement	Study design (RCT vs cohort studies)	No	A single study provided outcome data, which prevented subgroup analysis.[31]
		Gender	No	No included study reported sex-specific outcome data, which prevented subgroup comparisons.
		Age group	No	No included study reported age-stratified outcome data, which prevented subgroup comparisons.
		BMI (normal weight, overweight and obese)	No	No included study reported BMI-stratified outcome data, which prevented subgroup comparisons.
		CT examination type (hepatic, oncologic, routine, multiphasic, uniphasic and venous /portal phase CT)		A single study provided outcome data, which prevented subgroup analysis.[31]

Supplementary Table S6: Summary of compliance with PRISMA guidelines

Section and Topic	Item #	Checklist item	Location where item is reported
**TITLE**			1
Title	1	Identify the report as a systematic review.	
**ABSTRACT**			
Abstract	2	See the PRISMA 2020 for Abstracts checklist.	1
**INTRODUCTION**			
Rationale	3	Describe the rationale for the review in the context of existing knowledge.	2
Objectives	4	Provide an explicit statement of the objective(s) or question(s) the review addresses.	2
**METHODS**			
Eligibility criteria	5	Specify the inclusion and exclusion criteria for the review and how studies were grouped for the syntheses.	3
Information sources	6	Specify all databases, registers, websites, organisations, reference lists and other sources searched or consulted to identify studies. Specify the date when each source was last searched or consulted.	3
Search strategy	7	Present the full search strategies for all databases, registers and websites, including any filters and limits used.	Supplementary file S2

Section and Topic	Item #	Checklist item	Location where item is reported
Selection process	8	Specify the methods used to decide whether a study met the inclusion criteria of the review, including how many reviewers screened each record and each report retrieved, whether they worked independently, and if applicable, details of automation tools used in the process.	3
Data collection process	9	Specify the methods used to collect data from reports, including how many reviewers collected data from each report, whether they worked independently, any processes for obtaining or confirming data from study investigators, and if applicable, details of automation tools used in the process.	3
Data items	10a	List and define all outcomes for which data were sought. Specify whether all results that were compatible with each outcome domain in each study were sought (e.g. for all measures, time points, analyses), and if not, the methods used to decide which results to collect.	3
	10b	List and define all other variables for which data were sought (e.g. participant and intervention characteristics, funding sources). Describe any assumptions made about any missing or unclear information.	3
Study risk of bias assessment	11	Specify the methods used to assess risk of bias in the included studies, including details of the tool(s) used, how many reviewers assessed each study and whether they worked independently, and if applicable, details of automation tools used in the process.	3
Effect measures	12	Specify for each outcome the effect measure(s) (e.g. risk ratio, mean difference) used in the synthesis or presentation of results.	4
Synthesis methods	13a	Describe the processes used to decide which studies were eligible for each synthesis (e.g. tabulating the study intervention characteristics and comparing against the planned groups for each synthesis (item #5)).	4
	13b	Describe any methods required to prepare the data for presentation or synthesis, such as handling of missing summary statistics, or data conversions.	4
	13c	Describe any methods used to tabulate or visually display results of individual studies and syntheses.	N/A
	13d	Describe any methods used to synthesize results and provide a rationale for the choice(s). If meta-analysis was performed, describe the model(s), method(s) to identify the presence and extent of statistical heterogeneity, and software package(s) used.	4-5
	13e	Describe any methods used to explore possible causes of heterogeneity among study results (e.g. subgroup analysis, meta-regression).	4-5
	13f	Describe any sensitivity analyses conducted to assess robustness of the synthesized results.	4-5
Reporting bias assessment	14	Describe any methods used to assess risk of bias due to missing results in a synthesis (arising from reporting biases).	3
Certainty assessment	15	Describe any methods used to assess certainty (or confidence) in the body of evidence for an outcome.	5
**RESULTS**			
Study selection	16a	Describe the results of the search and selection process, from the number of records identified in the search to the number of studies included in the review, ideally using a flow diagram.	5
	16b	Cite studies that might appear to meet the inclusion criteria, but which were excluded, and explain why they were excluded.	N/A
Study characteristics	17	Cite each included study and present its characteristics.	5

Section and Topic	Item #	Checklist item	Location where item is reported
Risk of bias in studies	18	Present assessments of risk of bias for each included study.	Supplemental file S1
Results of individual studies	19	For all outcomes, present, for each study: (a) summary statistics for each group (where appropriate) and (b) an effect estimate and its precision (e.g. confidence/credible interval), ideally using structured tables or plots.	10-14
Results of syntheses	20a	For each synthesis, briefly summarise the characteristics and risk of bias among contributing studies.	Tables 1
	20b	Present results of all statistical syntheses conducted. If meta-analysis was done, present for each the summary estimate and its precision (e.g. confidence/credible interval) and measures of statistical heterogeneity. If comparing groups, describe the direction of the effect.	10-14
	20c	Present results of all investigations of possible causes of heterogeneity among study results.	10-14
	20d	Present results of all sensitivity analyses conducted to assess the robustness of the synthesized results.	10-13
Reporting biases	21	Present assessments of risk of bias due to missing results (arising from reporting biases) for each synthesis assessed.	N/A
Certainty of evidence	22	Present assessments of certainty (or confidence) in the body of evidence for each outcome assessed.	Table 3
**DISCUSSION**			
Discussion	23a	Provide a general interpretation of the results in the context of other evidence.	17-18
	23b	Discuss any limitations of the evidence included in the review.	19
	23c	Discuss any limitations of the review processes used.	19
	23d	Discuss implications of the results for practice, policy, and future research.	19
**OTHER INFORMATION**			
Registration and protocol	24a	Provide registration information for the review, including register name and registration number, or state that the review was not registered.	N/A
	24b	Indicate where the review protocol can be accessed, or state that a protocol was not prepared.	N/A
	24c	Describe and explain any amendments to information provided at registration or in the protocol.	N/A
Support	25	Describe sources of financial or non-financial support for the review, and the role of the funders or sponsors in the review.	20
Competing interests	26	Declare any competing interests of review authors.	20
Availability of data, code and other materials	27	Report which of the following are publicly available and where they can be found: template data collection forms; data extracted from included studies; data used for all analyses; analytic code; any other materials used in the review.	N/A
